# Computational Analysis of Movement Patterns of Dogs with ADHD-Like Behavior

**DOI:** 10.3390/ani9121140

**Published:** 2019-12-13

**Authors:** Stephane Bleuer-Elsner, Anna Zamansky, Asaf Fux, Dmitry Kaplun, Sergey Romanov, Aleksandr Sinitca, Sylvia Masson, Dirk van der Linden

**Affiliations:** 1 Information Systems Department, University of Haifa, Haifa 3498838, Israel; vetbehavior.il@gmail.com (S.B.-E.); asaffoox@gmail.com (A.F.); 2 Department of Automation and Control Processes, Saint Petersburg Electrotechnical University “LETI”, Saint Petersburg 197376, Russia; dikaplun@etu.ru (D.K.); saromanov@etu.ru (S.R.); amsinitca@etu.ru (A.S.); 3 Clinique de la Tivolliere, 38340 Voreppe, France; S.masson@hotmail.com; 4 Department of Computer Science, University of Bristol, Bristol BS8 1TH, UK; djt.vanderlinden@gmail.com

**Keywords:** clinical behavior, computational analysis, dog hyperactivity, dog ADHD-like behavior

## Abstract

**Simple Summary:**

ADHD-like (attention deficit hyperactivity disorder) behavior in dogs may be expressed as impulsivity, inattentiveness, or aggression, compromising both dog and owner quality of life. Its treatment in a clinical setting requires behavioral modification and sometimes a medical treatment is added. There is a lack of objective tools for assessment and diagnosis of the problem, and behavioral experts mostly rely on owner reports. To address this gap, in this paper we use a self-developed computational tool which automatically analyzes movement of a dog from video footage collected during behavioral consultation. Based on a computational analysis of behavioral consultations of 12 dogs medically treated due to ADHD-like behavior and of a control group of 12 dogs with no reported behavioral problems, we identify three dimensions of characteristic movement patterns of dogs with ADHD-like behaviors, which are detectable during consultation. These include (i) high speed of movement, (ii) large coverage of room space, and (iii) frequent re-orientation in room space. These patterns can form the basis for computational methods for objective assessment of dogs with ADHD-like behavior that could help for diagnosis and clinical treatment of the disorder.

**Abstract:**

Computational approaches were called for to address the challenges of more objective behavior assessment which would be less reliant on owner reports. This study aims to use computational analysis for investigating a hypothesis that dogs with ADHD-like (attention deficit hyperactivity disorder) behavior exhibit characteristic movement patterns directly observable during veterinary consultation. Behavioral consultations of 12 dogs medically treated due to ADHD-like behavior were recorded, as well as of a control group of 12 dogs with no reported behavioral problems. Computational analysis with a self-developed tool based on computer vision and machine learning was performed, analyzing 12 movement parameters that can be extracted from automatic dog tracking data. Significant differences in seven movement parameters were found, which led to the identification of three dimensions of movement patterns which may be instrumental for more objective assessment of ADHD-like behavior by clinicians, while being directly observable during consultation. These include (i) high speed, (ii) large coverage of space, and (iii) constant re-orientation in space. Computational tools used on video data collected during consultation have the potential to support quantifiable assessment of ADHD-like behavior informed by the identified dimensions.

## 1. Introduction

There is an increasing interest in “objectivization” of behavior assessment methods. As Karen Overall noted: “A review of behavioral data over the past decade supports a serious shift to crisper definitions of terms and quantifiable assessment of behaviors. Veterinary behavior and veterinary behavioral medicine are coming later to this approach, but the change is welcome” [1].

One particularly relevant challenge in this context is the problem of assessment of dog ADHD-like (attention deficit hyperactivity disorder) behaviors. They are often expressed in the form of inattention, impulsivity, and aggressivity, greatly compromising life quality of the dog and its owner [2,3,4]. In addition, recently its links to human ADHD were explored; in particular, a pilot study by Puruunen et al. [5] identified associations between canine ADHD-like behaviors and metabolites involved in lipid and tryptophan metabolisms, which share similarity with earlier findings in human ADHD models.

Several assessment instruments relevant to ADHD-like behavior and its expressions such as impulsivity were considered in the literature. These include C-BARQ [6], the dog ADHD rating scale [3], Dog Impulsivity Assessment Scale (DIAS) [7], and other questionnaires and scales [2,4]. However, all of these scales are owner administered and thus may be affected by non-objectivity and inaccuracy (e.g., there is evidence that owners may misinterpret the behavior of their pets [8]).

Computational behavior analysis may offer new tools for more objective assessment of behavior in clinical settings. It is an emerging field encapsulating the use of modern methods from computer science and engineering to quantitatively measure animal behavior [9]. In this paper we use computational analysis in the context of clinical assessment of ADHD-like behavior as part of an ongoing study for developing a decision support system for behavioral clinicians (some first results already appeared in [10,11,12,13]).

Our hypothesis in this paper is that dogs with ADHD-like behavior may exhibit distinguishable movement patterns during consultation. To investigate the hypothesis, we compare a set of movement parameters (e.g., average speed, number of turns, and other trajectory characteristics) automatically extracted from video footage of behavioral consultations of 12 dogs treated due to ADHD-like behavior (we henceforth refer to this group as H-group) and of a control group (C-group) of 12 dogs with no known behavioral problems.

## 2. Materials and Methods

### 2.1. Participants

Twenty-four dogs were selected for the study. The C-group (N = 12, 5 males, 7 females) had a median age of 2.75 years (±2.15, max 7 years, min 8 months), median weight of 22.00 kg (±6.70, max 36, min 13), while the H-group (N = 12, 7 males, 5 females) had a median age of 1.50 years (±2.06, max 7 years, min 6 months), median weight of 20.58 kg (±8.16, max 34, min 12) (see Table 1 for details).

To rule out confounding effects of demographic factors on the experimental variables we ensured that the C-group was matched to H-group in terms of demographic factors. To verify this, we checked that there was no statistically significant difference between the two groups for any demographic factor: age did not differ significantly between the two groups (Mann–Whitney U test, U = 62, n1 = n2 = 24, *p* = 0.58 (two-tailed)), nor did weight (Mann–Whitney U test, U = 62, n1 = n2 = 24, *p* = 58 (two-tailed)). Categorical factors of sex (Fisher’s exact test, *p* = 0.56 (two tailed), φ = 0.31) and neutered state (Fisher’s exact test, *p* = 0.32 (two tailed), φ = −0.40) also did not differ significantly between the groups.

All subjects in the H-group were clinical patients of two behavioral veterinary clinics “Veterinar Toran Tel-Aviv” and “Veterinar Toran Petach Tikva” in Israel and were diagnosed with ADHD-like behavior. They were recorded at the time of their first visit to the clinic of the first author, a practicing behavioral veterinarian. Thus, the inclusion criteria for the H-group were the following:

1. This was the subjects’ first visit to the behavioral expert veterinarian.
2. During that visit the expert diagnosed the subject with ADHD-like behavior.
3. During that visit the expert prescribed a medical treatment for the above condition.

All subjects in the control group were dogs with no reported behavioral problems or issues. They were recruited in the two clinics “Veterinar Toran Tel-Aviv” and “Veterinar Toran Petach Tikva” in Israel during standard checkup and vaccination, or through the authors’ professional connections. The behavioral veterinarian ruled out any behavioral disorder during a consultation.

For all dogs from both groups it was their first visit to the consultation room. The owner-dog dyad entered the room and the dog was immediately released, at which point the recording was started.

### 2.2. Location

**Figure 1:**
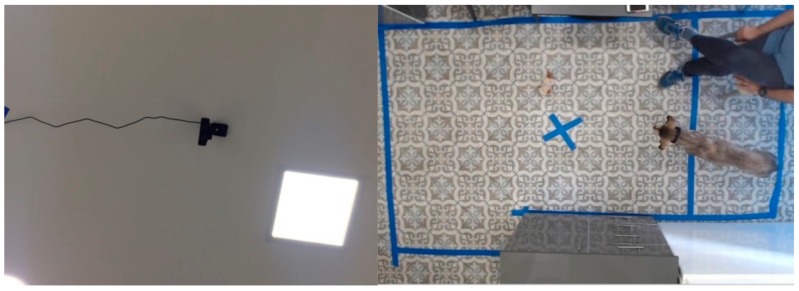
Camera fixed on the ceiling of clinic and an example frame taken by it.

Dogs were observed in the veterinary behavioral clinics at the Petah Tikva and Tel Aviv Animal Hospitals. The testing space captured by a camera fixed on the ceiling was 100 × 65 cm and 300 × 160 cm respectively. (To rule out confounding effects from the difference in floor size in the different clinics, we verified whether there was any significant difference in any measured variable between recording from the Tel Aviv (N = 18) and Petah Tikva (N = 6) clinic. A two-tailed Mann–Whitney U test found no significant difference for any of the variables (*p* > 0.05).) (see Figure 1). The dog owners sat at a fixed place outside of the captured frame.

### 2.3. Analysis Software

**Figure 2:**
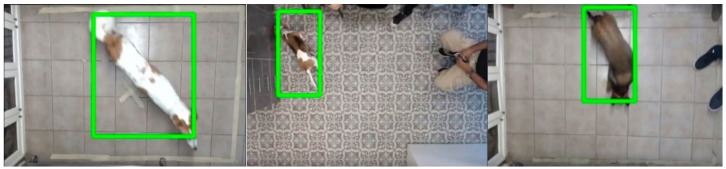
Example frames of dogs tracked in the clinics by K9-Blyzer.

We used a self-developed software tool, K9-Blyzer (first presented in Amir et al. [10] and later extended in [11,12,13]) for analyzing the video footage recorded at the clinics and producing the data on the movement variables discussed below. The system takes as input raw video of a dog freely moving in a room and produces as output the dog’s location (x,y) in each frame. Due to different distances of the camera from floor in the two clinics, calibration specific to clinic was applied to convert distances in pixels to centimeters. The system is based on a convolutional neural network which was trained on 6000 annotated frames of dogs in the two clinics. For frames where the dog is clearly visible, the system reaches 98% detection accuracy. Figure 2 shows some example frames where the tool tracks the dog.

### 2.4. Processed Videos

For each participant, the first three minutes of the consultation were taken for the computational analysis. Videos where the dog was not clearly identified in more than 30% of the frames were excluded from the analysis (the number of excluded dogs was 5).

### 2.5. Analyzed Parameters

In addition to basic movement related parameters: distance and average speed (that were previously used in the context of a vet consultation room [11]), we used five metrics known as animal movement indices in the literature: Alamedia et al. [14] mention intensity of use, straightness, sinuosity, mean squared displacement, and fractal dimension. Table 2 below provides explanations for each metric. We further added two more metrics intuitively related to “erratic” movement of an impulsive dog: number of turns the dog made, divided into four groups according to angle sharpness and number of approximation points on the curve of the dog’s trajectory, obtained by applying a variant of Ramer–Douglas–Pecker curve approximation algorithm [15].

## 3. Results

Analysis of the difference in measured variables between H-group and C-group using Mann–Whitney U test (given small sample sizes and non-normally distributed range of variables) revealed several statistically significant differences, as summarized in Table 3. In order to account for confounding effects of dog size on movement (e.g., larger dog breeds may cover more absolute distance than smaller dog breeds by nature of their size) we used an approximated normalization for the relevant parameters affected here (distance, average_speed) by dividing them by weight. We calculated the statistical power of each test to account for low sample size, disregarding any identified distinctions with a statistical power < 0.80. In particular, distance (normalized) and average_speed (normalized) revealed a significant difference between groups (*p* < 0.01, resp. Cohen’s d = 1.28, 1.45), as did most of the turn variables (30_60, 60_90, and 90_120; *p* < 0.01, Cohen’s d resp. 1.42, 1.64 and 1.28), number of points (*p* < 0.01, Cohen’s d 1.43), straightness (*p* < 0.05, Cohen’s d −1.24), and fractal dimension (*p* < 0.01, Cohen’s d 1.96).

## 4. Discussion

The parameters found significant by our analysis can be related to three dimensions for further exploration in the context of quantification and objectivization of assessment of ADHD-like behavior: (i) speed (as induced by average_speed parameter), (ii) coverage of room space (as induced by distance and FD), and (iii) reorientation in space (as induced by num_of_points, straightness, and the turn parameters).

It seems that the parameter of intensity of use is also strongly relevant to the second dimension. Indeed, while they were not found significant using the widely accepted minimal threshold of 0.8 for statistical power, they were close to it, and further experimentation with additional samples may highlight a different picture.

The identified dimensions of distance and coverage of space support the suggested links of ADHD-like behavior to increased motor activity [4] and lack of stop signal in the behavioral sequence and low threshold of sensorial homeostasis [2]. High frequency of re-orientation may also be explained by the link to inattentiveness [4] and strong reaction to the lightest stimuli [2].

These dimensions can form the basis for further developing computer-based instruments for quantified assessment of ADHD-like behavior. Eventually their envisioned use is for decision support system for behavioral clinicians in diagnosis and treatment of behavioral problems and disorders. It should be noted, however, that the current study considered only the differences between dogs with pure ADHD-like behavior to normal dogs, so no conclusions about the specificity of the system can be made at this stage, hence dogs with other behavioral problems, such as anxiety, may also exhibit similar patterns. This remains to be tested and is one immediate direction for future research.

Moreover, the above-mentioned dimensions are not expected to replace owner-reported information but complement it in new objective ways. Another future research direction we are already involved in is integrating a web-based decision support system into the behavioral vet’s workflow, which analyzes video footage in real-time, providing the different parameters values and visualizing them. An additional research direction will be further validation of the video-tracking system using the various owner-reported questionnaires mentioned above, which continue to provide the common practice for an assessment of canine ADHD-like behavior. Finally, further data collection for a more in-depth comparison with respect to groups of dogs diagnosed with other behavioral disorders (such as anxiety or depression) is another direction we plan to explore.

## Figures and Tables

**Table 1:** Participants’ demographic information.

**C-Group (N = 12)**					
**Name**	**Sex (m/f)**	**Age (yrs)**	**Breed**	**Weight (kgs)**	**Neutered**
Wally	m	3	Saluki	23	y
Gino	m	0.67	Cane Corso	44	n
Jema	f	3	Mixed	25	y
Laila	f	1	Mixed	20	y
Loli	f	6	Mixed	17	y
Misty	f	2.5	Mixed	20	y
Pie	m	1	Mixed	25	y
Sparki	m	5	Golden Retriever	40	n
Mila	f	3.5	Mixed	22	y
Mika	f	7	Mixed	7	y
Theresa	f	0.67	Saluki	16.5	y
Pit	m	1	Mixed	25	y
**H-Group (N = 12)**					
**Name**	**Sex (m/f)**	**Age (yrs)**	**Breed**	**Weight (kgs)**	**Neutered**
Sia	f	2	Mixed	19	y
Tomy	m	2.5	French Bulldog	13	y
Humus	m	1.5	Mixed	23	y
Indi	f	1.5	Vizsla	20	y
Max	m	1	Labrador	36	y
Pit	m	3	Mixed	24	y
Nancy	m	0.67	Mixed	21	y
Bana	f	1.5	Doberman	32	y
Patrick	m	0.5	Husky	23	n
Mitch	m	6	French Bulldog	13	n
Lichi	f	7	Mixed	22	y
Kim	f	1	Mixed	18	y

**Table 2:** Description of analyzed variables.

Variable	Explanation	Unit
n-distance	Distance covered by the dog normalized by its weight	cm
turn30_60	Number of turns between 30 and 60 degrees	
turn60_90	Number of turns between 60 and 90 degrees	
turn90_120	Number of turns between 90 and 120 degrees	
turn120	Number of turns greater than 120 degrees	
IU	(Intensity of Use) the ratio between total movement and the square root of the area of movement	Percentage
number_of_points	Number of approximation points on the curve resulting from applying a variant of Dougles–Peuker curve approximation algorithm to the dog’s trajectory	
average_speed	Dog’s walking distance in relation to calculated video duration	cm/s
ST	Straightness-net displacement distance divided by the total length of the dog’s movement	Varies from 0 to 1
MSD	Mean squared displacement—measure of the deviation of the position of a particle with respect to the dog’s reference position over time	cm^2^ s^−1^
SI	Sinuosity—calculattion of the actual path length divided by the shortest path length of the dog’s movement	Varies from 1 to infinity
FD	Fractal Dimension—statistical index ratio of complexity comparing the space-filling capacity of the dog’s movement pattern	

**Table 3:** Observed values for analyzed variables and differences between groups.

Variable	H-Group		C-Group		Mann–Whitney U Test of Independence (n_1_ = n_2_)			
	Mean	SD	Mean	SD	U	*p*	Effect Size (Cohen’s d)	Power (posthoc)
n-distance	214.88	169.63	57.79	38.81	15	<0.01	1.28	0.83
turn30_60	27.00	14.85	11.08	5.48	24	<0.01	1.42	0.90
turn60_90	12.08	6.75	3.42	3.20	12	<0.01	1.64	0.96
turn90_120	7.75	5.55	2.17	2.72	18.5	<0.01	1.28	0.83
turn120	20.92	21.90	6.00	5.38	29	<0.05	0.94	0.57
IU	25.50	19.66	9.13	4.88	17	<0.01	1.14	0.74
number_of_points	217.42	138.43	73.33	34.92	14	<0.01	1.43	0.90
average_speed (normalized)	1.49	1.08	0.36	0.23	10	<0.01	1.45	0.91
ST	0.02	0.03	0.10	0.09	16	<0.05	−1.24	0.81
MSD	198.65	62.51	231.12	195.05	67	=0.77	−0.22	0.08
SI	0.33	0.09	0.39	0.08	43	=0.1	−0.68	0.34
FD	1.40	0.11	1.20	0.10	14	<0.01	1.96	0.99
